# Gasdermin D protects against *Streptococcus equi subsp. zooepidemicus* infection through macrophage pyroptosis

**DOI:** 10.3389/fimmu.2022.1005925

**Published:** 2022-10-14

**Authors:** Guobin Xu, Zheng Guo, Yuxuan Liu, Yalin Yang, Yongjin Lin, Chunliu Li, Yunfei Huang, Qiang Fu

**Affiliations:** ^1^ School of Life Science and Engineering, Foshan University, Foshan, China; ^2^ Foshan University Veterinary Teaching Hospital, Foshan University, Foshan, China

**Keywords:** GSDMD, pyroptosis, SEZ infection, macrophage, IL-1β, IL-18

## Abstract

*Streptococcus equi subsp. zooepidemicus* (*S. zooepidemicus*, SEZ) is an essential zoonotic bacterial pathogen that can cause various inflammation, such as meningitis, endocarditis, and pneumonia. Gasdermin D (GSDMD) is involved in cytokine release and cell death, indicating an important role in controlling the microbial infection. This study investigated the protective role of GSDMD in mice infected with SEZ and examined the role of GSDMD in peritoneal macrophages in the infection. GSDMD-deficient mice were more susceptible to intraperitoneal infection with SEZ, and the white pulp structure of the spleen was seriously damaged in GSDMD-deficient mice. Although the increased proportion of macrophages did not depend on GSDMD in both spleen and peritoneal lavage fluid (PLF), deficiency of GSDMD caused the minor release of the pro-inflammatory cytokines interleukin-1β (IL-1β) and interleukin-18 (IL-18) during the infection *in vivo*. *In vitro*, SEZ infection induced more release of IL-1β, IL-18, and lactate dehydrogenase (LDH) in wild-type macrophages than in GSDMD-deficient macrophages. Finally, we demonstrated that pore formation and pyroptosis of macrophages depended on GSDMD. Our findings highlight the host defense mechanisms of GSDMD against SEZ infection, providing a potential therapeutic target in SEZ infection.

## Introduction

*Streptococcus equi subsp. zooepidemicus* (SEZ), a Gram-positive *Streptococcus*, belongs to beta-hemolytic *Streptococcus* of the Lancefield group C. SEZ could lead to infection in various mammals, including humans (1, 2). According to reports in recent years, SEZ has caused not only a high mortality rate of pigs in many countries and regions, such as the USA and Canada (3, 4), but also infection in humans, including pneumonia, meningitis, and even death (5, 6). Previous studies suggested that SEZ could invade human epithelial cells and cause infection (7), which motivates us to focus on the risks of SEZ infection in humans and animals. Significant progress in vaccine research against SEZ infection has been facilitated (8), especially in SEZ surface proteins (9). However, how the innate immune system protects the host against SEZ is still need for further study. Moreover, macrophages serve as the first responders to the immune system. They can effectively defend against microbial invasion, and others have demonstrated that NLRP3 inflammatory and caspase-1 in macrophages were vitally protective against bacterial infections (10, 11).

Pyroptosis, an emerging pro-inflammatory form of lytic cell death, is triggered when the pro-inflammatory caspases (caspase-1, -4, -5, and -11) are activated (12, 13), and GSDMD was defined as an executor of pyroptosis. Once pathogens invasion is detected, GSDMD will be cleaved by pro-inflammatory caspases by canonical and non-canonical inflammasome pathways (14, 15). However, more pyroptosis about hazards of excessive inflammation was reported (16). As we know, inflammation is a double-edged sword, and the protective role of pyroptosis still needs further study in SEZ infection.

GSDMD is an important member of the GSDM protein family, its C-terminal structural domain (GSDMD-C) can inhibit the N-terminal (GSDMD-N) activity before GSDMD cleavage, and there is self-inhibition. After cleavage, its N-terminal structural domain activity will not be inhibited and be aggregated on the membrane to form the 10-20 nm pore (15, 17, 18), triggering cell pyroptosis subsequently. In addition, GSDME in the GSDM protein family can also undergo similar cleavage and trigger pyroptosis (19, 20), suggesting that GSDMD is not the only protein to trigger pyroptosis. However, the role of GSDMD in SEZ infection needs further investigation, and whether SEZ infection can induce pyroptosis remains unclear. As the study has shown, GSDMD deficiency reduces the secretion of IL-1β and IL-18 in not all infections (21), so whether GSDMD can promote the protective inflammatory triggered by pro-inflammatory cytokines (such as IL-1β and IL-18) *in vivo* after SEZ infection remains to be further studied.

## Materials and methods

### Ethics statement

All the animal experiments described in the current study were conducted in strict accordance with the guidelines of the National Guidelines for Experimental Animal Welfare. The animal study was reviewed and approved by the laboratory animal Monitoring Committee of Guangdong Province and performed accordingly (approval number SYXK (Guangdong) 2019-0136). All efforts were made to minimize suffering and ensure the highest ethical and humane standards.

### Mice

Wild-type (GSDMD^+/+^) and GSDMD knockout (GSDMD^-/-^ ) mice are obtained from the Nanjing Biomedical Research Institute of Nanjing University (Nanjing, China). On C57BL/6 genetic background, all mice were bred under specific pathogen-free conditions in the Animal Center of South China Agricultural University (Guangzhou, China). All experiments used age (6- to 8-week-old) and sex-matched animals.

### Bacteria strains, intraperitoneal infection, and treatments

SEZ strain C55138 (China Institute of Veterinary Drug Control) was isolated from a septic pig and preserved in our laboratory (22). The strains were isolated to single colonies on the Tryptose Soya Agar (TSA; Oxoid, UK) medium containing 5% newborn calf serum. Then Tryptone Soybean Broth (Oxoid, UK) containing 5% newborn calf serum was used to culture the SEZ at 37°C for 10 h to the midlogarithmic phase. The concentration of bacteria in the media was determined *via* plating serial dilutions on TSA. The bacterial solution was diluted with sterile PBS to the indicated titer. For mice infection, mice were inoculated intraperitoneally with SEZ 1×10^6^ colony-forming units (CFU) in 500 μL PBS, and the same volume of sterile PBS was intraperitoneally injected into mice as the control group. To record the survival rate, all mice were monitored three times daily for their clinical signs during the 10 days of SEZ infection. The clinical scores were assigned as follows: 0 = normal response to stimuli; 1 = ruffled coat and slow response to stimuli; 2 = respond only to repeated stimuli; 3 = non-responsive or walking in circles; and 4 = dead. Mice losing 20 percent of their pre-experiment weight or exhibiting extreme lethargy or neuro- logical signs (score = 3) were considered moribund and were humanely euthanized (22). The mortality of the two genotypes of mice was calculated. In this assessment, the experimenters were blinded to GSDMD^+/+^ mice versus GSDMD^−/−^ mice.

### Hematoxylin and eosin staining

Three GSDMD^+/+^ mice and three GSDMD^-/-^ mice were injected intraperitoneally with SEZ (10^6^ CFU) for 24 h, and the same amounts of mice were treated with the equivalent volume of PBS. The spleen of mice was removed and subsequently treated with 4% paraformaldehyde for 24 h. As described by others, spleen tissue was sliced into 4-μm-thick sections after paraffin embedding (23). The sections were dewaxed, gradient ethanol hydrated, and finally stained with hematoxylin-eosin (HE; Servicebio, China).

### Isolation of mouse peritoneal macrophages

Peritoneal macrophages were isolated from the untreated and healthy mice, referring to previously reported methods (24, 25). Briefly, the mice were sacrificed, 5 mL of sterile PBS was injected intraperitoneally, then the abdomen was massaged gently for 3 min, and the peritoneal lavage fluid (PLF) was finally drawn back. Next, cells in the PLF were collected through centrifugation at 200×g for 10 min. Then, the cells were cultured in DMEM (Gibco, USA) which has supplemented with 10% Fetal Bovine Serum (FBS; Gibco, USA), 100 U/mL penicillin, and 100 U/mL streptomycins. The cells were seeded in 96 or 12 well plates subsequently. After incubation for 2 h at 37 °C in a humidified incubator containing 5% CO_2_, the cells were washed three times with PBS to remove non-adherent cells, and a freshly prepared medium was added. The remaining cells were defined as peritoneal macrophages.

### PLF collection and treatment, flow cytometry, and cytokine measurements

As previously described (25), PLF was collected from euthanized mice by intraperitoneal injection and aspiration of 1 ml PBS. Cells obtained from PLF were counted. PLF was detected by Auto Hematology Analyzer (BC-5000 Vet; Mindray, China) for the amount of monocyte. For macrophage counting, the cells from PLF were stained for 30 min with Anti-F4/80 (1:80, BM8, eBioscience, USA) and Anti-CD11b (1:100, M1/70, eBioscience, USA) after being blocked with Anti-CD16/CD32 (1:62, 93, eBioscience, USA), the result was obtained with a CytoFLEX flow cytometer (Beckman Coulter, USA). Software CytExpert 2.3 was used to analyze the data. Cytokine levels in PLF and macrophage supernatants were measured by ELISA using the IL-1β and IL-18 kits (Multi Sciences, China).

### Western blotting

Peritoneal macrophages incubated with or without SEZ were treated with RIPA lysis buffer to extract total proteins. Protein concentration was measured using BCA Protein Assay Kit (Beyotime Biotechnology, China). An equal amount of samples were separated by SDS-PAGE. All proteins were subsequently electrotransferred onto the polyvinylidene difluoride (PVDF) membranes and then blocked with 5% bovine serum albumin (BSA) for 1 h at room temperature. After washing three times, the PVDF membranes were incubated overnight at 4 °C with the following primary antibodies diluted in BSA: Anti-GSDMD/GSDMD-N antibody (1:1000, ab219800) and Anti-β-Actin antibody (1:1000, ab8227) from Abcam. After washing away remain primary antibodies, the membranes were incubated with second antibodies (Goat Anti-Rabbit IgG H&L, 1:10000, ab205718, Abcam, UK) at room temperature for 1 h, followed by visualization using an ECL reagent (Solarbio, China). Finally, Immunoblot detection was performed by a Gel Imaging System (4200; Tanon, China). All proteins were scanned and quantified by the ImageJ software.

### Lactate dehydrogenase (LDH) analysis

GSDMD^+/+^ and GSDMD^-/-^ macrophages from the PLF were obtained and purified as described above. These cells were separately seeded in a 96-well plate (2 × 10^5^ cells per well) and allowed to adhere for 2 h at 37 °C. After being untreated or treated with SEZ of a multiplicity of infection (MOI) of 10:1 for 12 h, the level of LDH in the supernatants was analyzed using an LDH cytotoxicity assay kit (Beyotime Biotechnology, China) according to the manufacturer’s instructions.

### Pore formation assay

Propidium iodide (PI), a DNA dye that cannot penetrate living cell membranes, is enabled to assess cell pore formation (26, 27). GSDMD^+/+^ and GSDMD^-/-^ macrophages were plated in 6 well plates separately (2×10^6^ cells per well). After untreated or treated with SEZ (MOI=10) for 12 h, cells were gently washed twice with sterile PBS, and then the PI dye was added to all wells for 5 min incubation. Finally, the fluorescence microscope was used to observe the PI-positive cell.

### Scanning electron microscopy

After being untreated or treated with SEZ for 12 h, macrophages were fixed with 1% osmium tetroxide for 40 min and washed with sterile PBS three times. Samples were dehydrated through a graded series of ethanol (30, 50, 70, 80, 90, and 100%) and freeze-dried using the tertiary butanol method. Dried specimens were sputter coated with gold-palladium and imaged with an emission scanning electron microscope (Hitachi S-3400 N, Japan) operating at 5 kV (28).

### Statistical analysis

All data were expressed as mean ± SD. Survival curves were compared using the log rank Kaplan–Meier test. One-way ANOVA was used to analyze data, as specified in the figure legends. Analyses were performed using GraphPad Prism version 8.0.2 (GraphPad Software, La Jolla, CA). *P <*0.05 was considered statistically significant.

## Results

### GSDMD is protective in SEZ infection

**Figure 1:**
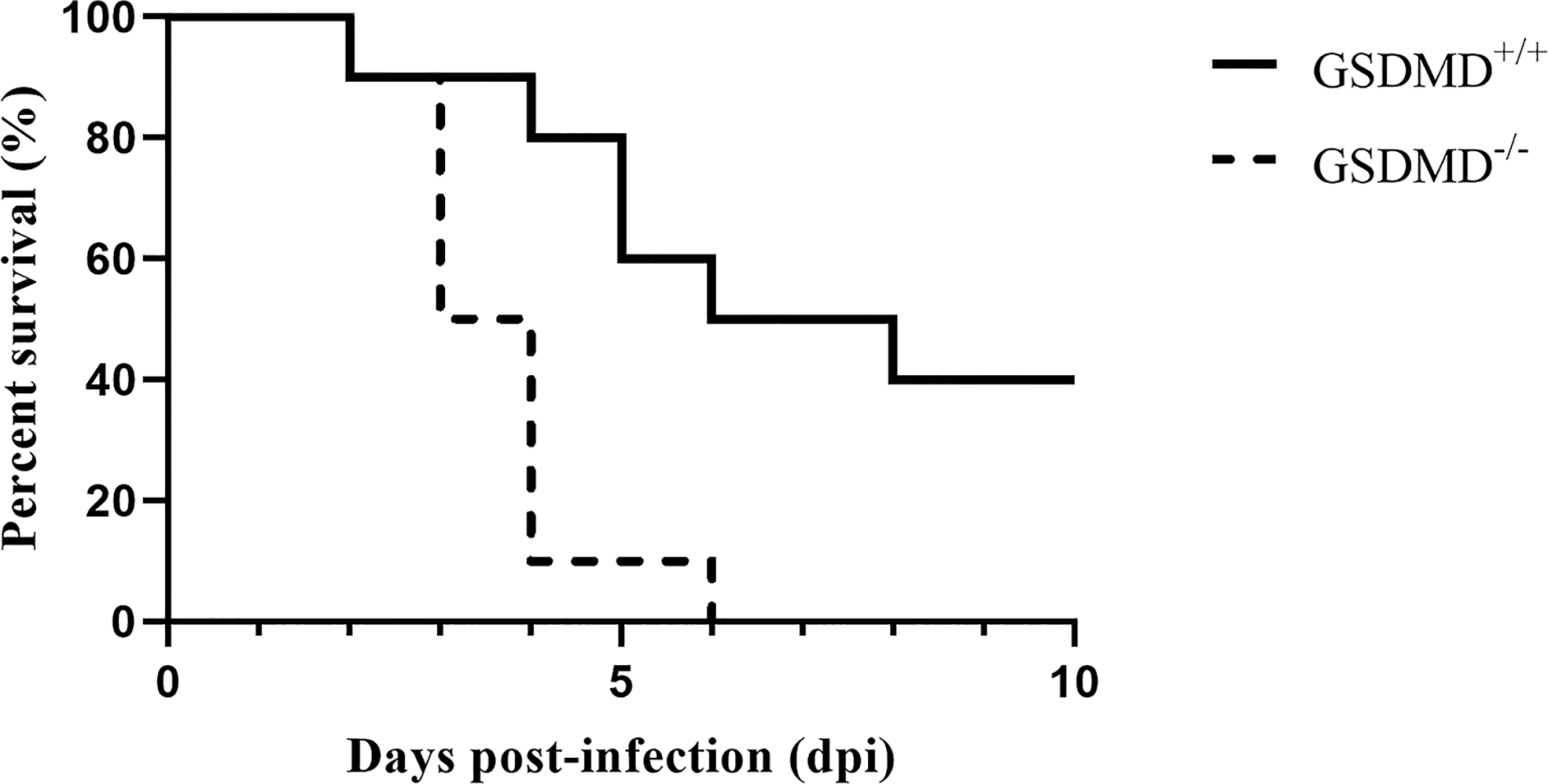
GSDMD is protective in SEZ infection. Mice were intraperitoneally injected with SEZ (10^6^ CFU), and their survival was monitored for 10 days (n=10 mice per group). The experiment was conducted with 3 replicates. The log rank Kaplan–Meier test was used for statistical analysis.

Previous studies suggested that GSDMD plays an essential role in some bacterial infections (29). We firstly verified whether GSDMD is indispensable against SEZ infection. As shown in **Figure 1**, GSDMD^-/-^ mice are more susceptible to SEZ. One GSDMD^-/-^ mice died on day 2 post-challenge, and the remaining GSDMD^-/-^ mice showed significant clinical signs such as a ruffled hair coat, a slow response to stimuli and died successively within 4 days. The death peak time of GSDMD^-/-^ mice was the third and fourth days after infection. Besides, one GSDMD^+/+^ mouse died on day 2 post-infection, the remaining mice showed mild clinical signs such as depression and weakness, and the death peak time of GSDMD^+/+^ mice was fourth to sixth days after infection. Moreover, all GSDMD^-/-^ mice had succumbed before the sixth day after infection, while 40% of GSDMD^+/+^ mice still survived at the end of the 10 days post-infection. These results indicated that GSDMD had effectively improved survival in mice after SEZ challenge.

### GSDMD deficiency results in enhanced pathology but not macrophages number in the spleen during SEZ infection

**Figure 2:**
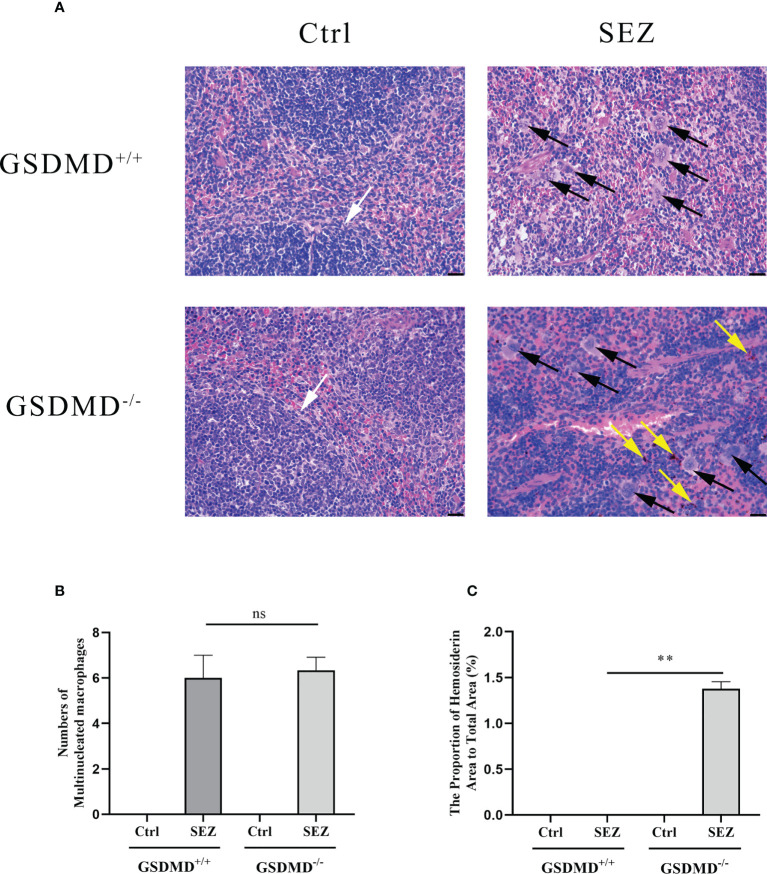
GSDMD deficiency results in enhanced pathology but not macrophages number in the spleen during SEZ infection. GSDMD^+/+^ and GSDMD^-/-^ mice (n=3) were treated with SEZ (10^6^ CFU) or equivalent PBS for 24 h. The black arrow marks the multinucleated macrophages. The yellow arrow indicates hemosiderin. The white arrow points to the clear white pulp of the spleen. One representative experiment of three is shown. Scale bar, 25 μm **(A, B)** The number of multinucleated macrophages was calculated in the microscopic fields of the slides. **(C)** The proportion of hemosiderin area to total area was calculated by randomly selecting three microscopic fields. These experiments were conducted with 3 replicates. Data are expressed as mean ± SD. ns, not significant, **p<0.01, one-way ANOVA **(B, C)**.

To better understand whether the pathological changes in the spleen can be observed after infection, the pathologic slices of the spleen are made with HE. Spleen from GSDMD^+/+^ and GSDMD^-/-^ mice showed clear signs of damaged white pulp structure after SEZ infection, but more hemosiderin could be observed in the spleen of GSDMD^-/-^ mice than in the spleen of GSDMD^+/+^ mice after the infection (**Figures 2A, C**). Meanwhile, more multinucleated macrophages were observed in the spleen of GSDMD^+/+^ and GSDMD^-/-^ mice after infection than in control (**Figure 2A**). Furthermore, there was no significant difference in the numbers of multinucleated macrophages between the GSDMD^+/+^ and GSDMD^-/-^ mice after infection (**Figure 2B**). These results suggested that GSDMD deficiency caused more splenic pathological changes rather than macrophage recruitment after SEZ infection.

### SEZ infection promotes the recruitment of macrophages in PLF

**Figure 3:**
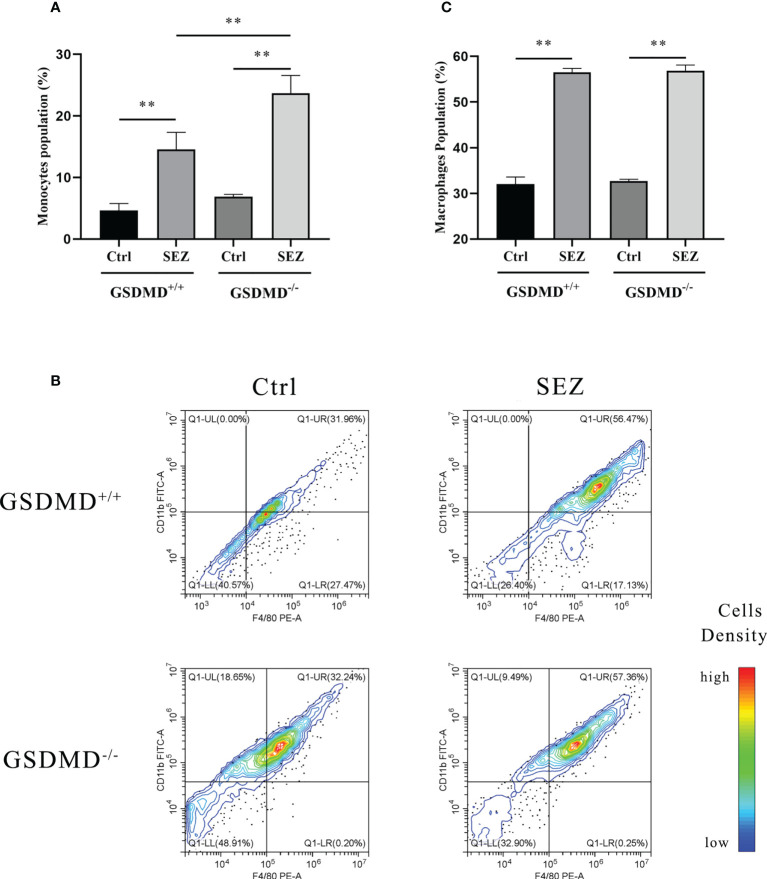
SEZ infection promotes the recruitment of macrophages in PLFv. **(A)** The number and proportion of monocytes collected from PLF of mice (n=5) were measured using Auto Hematology Analyzer, and **(B)** macrophages were assessed by F4/80/CD11b and analyzed by flow cytometry. A color scale bar was added to show the cells density (blue, green, and red represent low cell density, medium cell density, and high cell density, respectively). **(C)** The cell numbers in the upper right quadrant (F4/80-positive/CD11b-positive, macrophages) were counted and shown. All cells were isolated from mice infected or uninfected with SEZ (10^6^ CFU) for 24 h. These experiments were conducted with 3 replicates. Data are expressed as mean ± SD. **p<0.01, one-way ANOVA.

The PLF of mice was collected to evaluate the changes in the proportion of peritoneal monocytes and macrophages. After intraperitoneal injection of SEZ, the rate of monocytes in mice increased significantly in a GSDMD-dependent way. The proportion of monocytes in GSDMD^-/-^ mice was higher than in GSDMD^+/+^ mice (**Figure 3A**). The proportion of macrophages in the PLF was further analyzed by Flow cytometry. Compared with the control, the peritoneal macrophage proportion was increased significantly in GSDMD^+/+^ and GSDMD^-/-^ mice infected with SEZ, but there was no significant difference in peritoneal macrophage proportion shown between the GSDMD^+/+^ mice and GSDMD^-/-^ mice after the infection (**Figures 3B, C**). These results indicated that GSDMD deficiency significantly recruited more monocytes in mice challenged with SEZ than in control. However, macrophage number change in mice infected with SEZ was not dependent on GSDMD.

### GSDMD deficiency significantly reduces IL-1β and IL-18 release during SEZ infection

**Figure 4:**
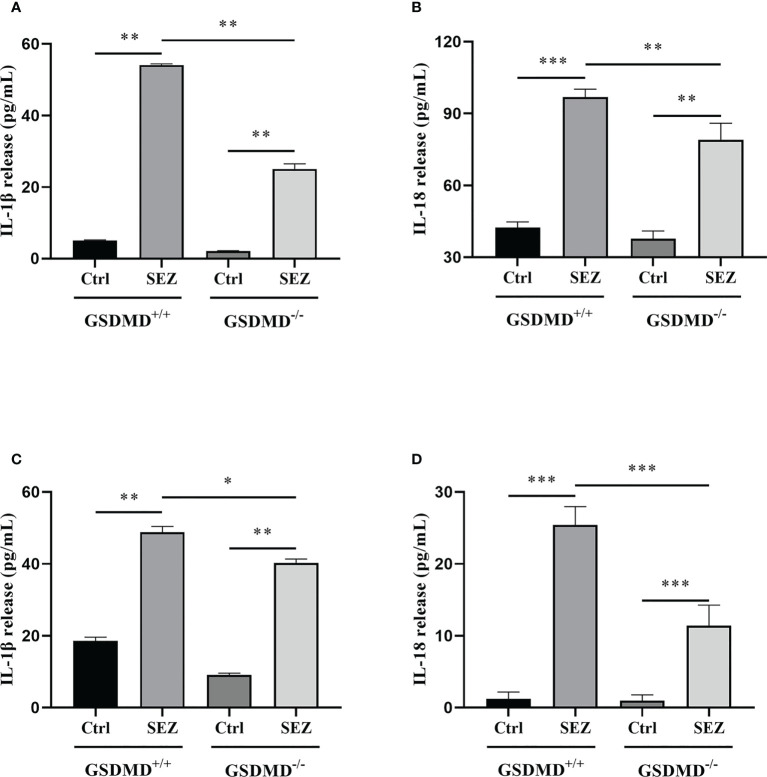
GSDMD deficiency significantly reduces IL-1β and IL-18 release during SEZ infection. **(A)** IL-1β and **(B)** IL-18 secretion in PLF of mice (n=3) were measured at 24 h post-infection (10^6^ CFU) or non-infection (equivalent PBS), and **(C)** IL-1β and **(D)** IL-18 release were determined in the macrophage supernatants with or without SEZ (MOI=10) treatment for 12 h. These experiments were conducted with 3 replicates. Data are expressed as mean ± SD. *p<0.05, **p<0.01, ***p<0.001, one-way ANOVA.

In order to test whether SEZ would trigger the secretion of IL-1β and IL-18 *in vivo*, cytokine levels in PLF were measured by enzyme-linked immunosorbent assay (ELISA) after 24h-infection. IL-1β and IL-18 release were increased remarkably after SEZ infection compared with the control, and the release of IL-1β and IL-18 depend on GSDMD (**Figures 4A, B**). Other research suggested that macrophages were responsible for cytokine release (30). Combined with the above results (**Figures 2A, B**), SEZ infection resulted in severe damage to the spleen and increased multinucleated macrophages and peritoneal macrophages in a GSDMD-independent way, so we postulated that peritoneal macrophages contribute to IL-1β and IL-18 release. When macrophages were stimulated with SEZ, the secretions of IL-1β and IL-18 were elevated. In contrast, GSDMD deficiency significantly decreases the release of IL-1β and IL-18 (**Figures 4C, D**), similar to the results in PLF (**Figures 4A, B**). These results suggest that GSDMD plays a crucial role in regulating IL-1β and IL-18 release after infection. Besides, the lack of GSDMD seems to be related to some function of macrophages rather than its recruitment.

### LDH release and expression of GSDMD-N in peritoneal macrophages are promoted after SEZ infection

**Figure 5:**
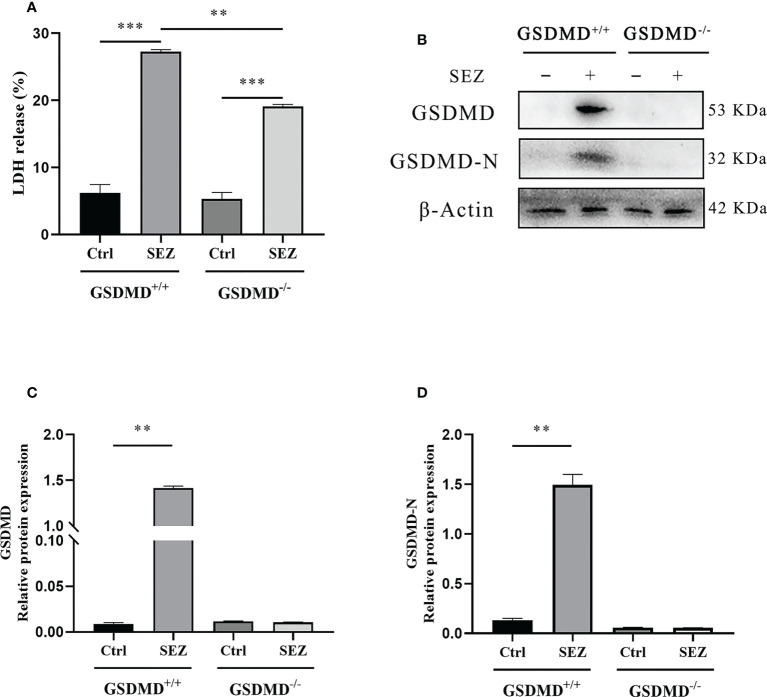
LDH release and expression of GSDMD-N in peritoneal macrophages are promoted after SEZ infection. **(A)** The release of LDH in culture supernatants was measured using an LDH cytotoxicity assay kit. **(B)** GSDMD and GSDMD-N proteins in GSDMD^+/+^ or GSDMD^-/-^ macrophages were performed by Western blotting. **(C, D)** The relative intensities are expressed as the ratio of relative proteins to β-Actin. These experiments were conducted with 3 replicates. One representative experiment of three **(B)** is shown. Data are expressed as mean ± SD. **p<0.01, ***p<0.001, one-way ANOVA **(A)**.

Except for the increased release of IL-1β and IL-18, pyroptosis was generally characterized as those pyroptosis-related proteins, including GSDMD, GSDMD-N, and LDH, significantly increased (31, 32). LDH release and cleavage of GSDMD are usually used to judge the occurrence of pyroptosis. In our study, the release of LDH in cell supernatant was increased in macrophages stimulated with SEZ. In contrast, GSDMD^-/-^ macrophages release less LDH (**Figure 5A**). In addition, the expression of GSDMD and GSDMD-N domain protein in macrophages is highly increased after SEZ infection, while these proteins are not expressed in GSDMD^-/-^ macrophages (**Figures 5B–D**). Taken together, SEZ can trigger macrophage GSDMD-mediated pyroptosis.

### Peritoneal macrophages have distinct morphological features of pyroptosis after SEZ infection

**Figure 6:**
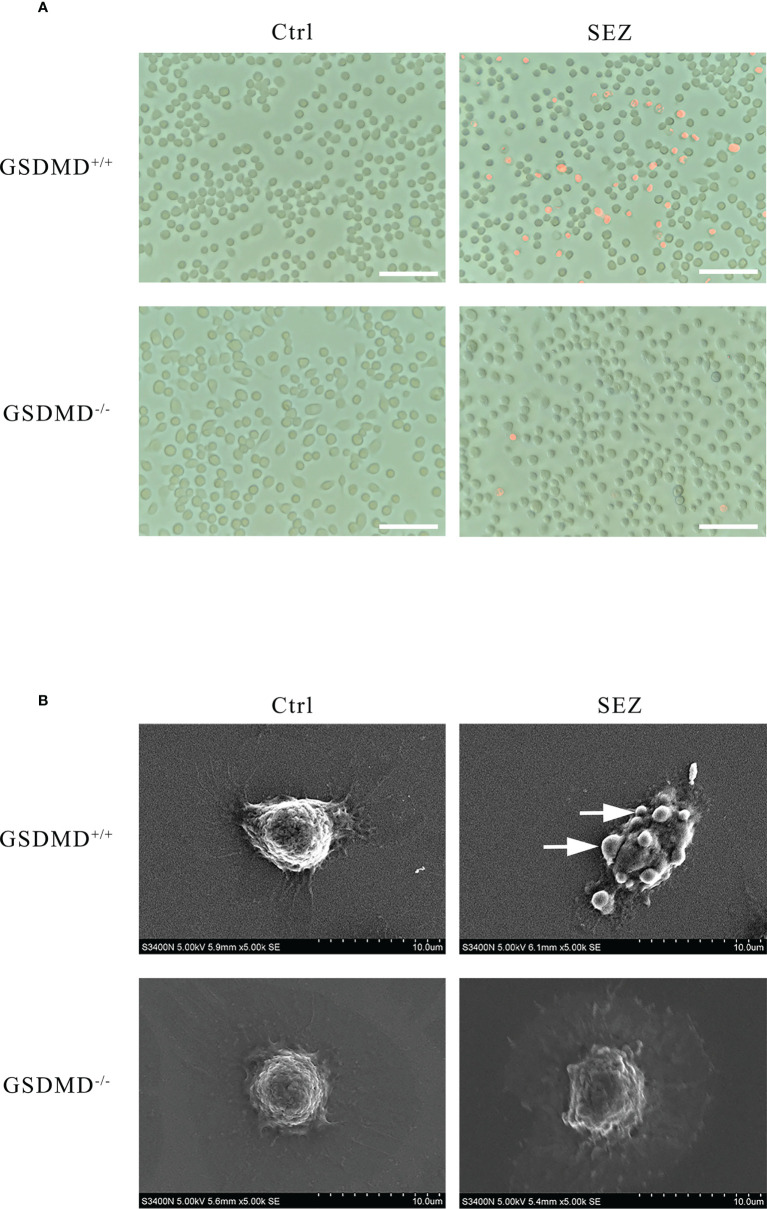
Peritoneal macrophages have distinct morphological features of pyroptosis after SEZ infection. **(A)** Representative propidium iodide (PI) staining of GSDMD^+/+^ or GSDMD^-/-^ macrophages after being infected or uninfected with SEZ. **(B)** Representative scanning electronic microscopy (SEM) images of GSDMD^+/+^ or GSDMD^-/-^ macrophage cells are treated as above, and the arrow points to the bubbling of pyroptotic cells. Scale bar, 70 μm **(A)**. These experiments were conducted with 3 replicates.

Propidium iodide (PI), a dye that cannot pass through the living cell membrane, is conducted to assess pore formation, which is critical for the research of pyroptosis (31, 33). When macrophages are stimulated with SEZ, more cells are stained red by Fluorescence Microscope than in control. GSDMD deficiency decreases the number of PI-positive cells after the infection (**Figure 6A**). Moreover, scanning electron microscopy is used to observe the ultrastructures of cells (28). The bubble-like protrusions of pyroptotic cells can be observed evidently in macrophages stimulated with SEZ but not in the untreated macrophages. On the contrary, the bubble-like protrusions of GSDMD^-/-^ macrophages cannot be observed after infection (**Figure 6B**). These results demonstrated that SEZ infection could induce macrophages pyroptotic bodies and pore-forming, which was dependent on GSDMD. These findings also support that macrophage GSDMD-mediated pyroptosis was induced after SEZ infection.

## Discussion

In recent years, SEZ caused a worldwide variety of diseases in various species, such as horses, pigs, sheep, dogs, and humans (4, 34–37). SEZ could infect humans or other animals through fecal transmission, food transmission, and contact transmission, threatening the health of humans and other animals with high mortality (4, 38, 39). However, prophylaxis and treatment of SEZ were not ideal (40). In addition, the use of antibiotics is subject to severe limitations, so new therapies for SEZ infection need to be developed. Previous studies have indicated that SEZ caused peritonitis which may further induce life-threatening sepsis (41–44). To better understand how the host responds to peritonitis caused by SEZ, we infected mice with SEZ by intraperitoneal injection (45). Intraperitoneal infection in experimental animals has been considered a suitable infection route, producing a high infection level (46). Previous studies found the crucial role of the inflammatory response and pyroptosis mediated by gasdermins in some diseases (17, 47–49). GSDMD was identified as the most likely pyroptosis effector activated in response to bacterial pathogens and other infectious pathogens, and some Gram-positive bacteria such as *Listeria monocytogenes* and *Staphylococcus aureus* have been studied in Gasdermin-mediated pyroptosis (50). Besides, according to the previous results in our laboratory, SEZ had hypervirulence to cause lethality in mice (25). In this study, we focus on the role of GSDMD in SEZ infection. Our research found that GSDMD^-/-^ mice were more susceptible to death by SEZ infection and had higher mortality than GSDMD^+/+^ mice (**Figure 1**). A similar protective effect of GSDMD also was found in Melioidosis (51). Some genes encoding virulence factors such as *ENuc* and *5Nuc* could reduce the function of host immune cells, which may be related to the high mortality of SEZ (52). Further studies are needed to determine how SEZ interacts with GSDMD. Furthermore, the spleen of GSDMD^-/-^ mice suffered greater damage than GSDMD^+/+^ mice after SEZ infection (**Figure 2A**). In addition to causing damage to the splenic white pulp, GSDMD deficiency caused hemosiderin deposition (**Figure 2A**), which usually indicated that red blood cell (RBC) hemolysis was induced (53). Our results suggested that GSDMD^-/-^ mice had RBC hemolysis after SEZ infection (**Figures 2A, C**).

Macrophages in the spleen are involved in innate immune responses *via* clearing bacteria and producing pro-inflammatory cytokines (54, 55). Our data found that SEZ infection resulted in the increase of multinucleated macrophages in the spleen (**Figures 2A, B**). In contrast, no significant difference in multinucleated macrophages number was shown between GSDMD^+/+^ and GSDMD^-/-^ mice infected with SEZ (**Figure 2B**). This result suggests that GSDMD deficiency cannot diminish multinucleated macrophages number in the spleen after SEZ infection. Previous studies suggested there is a close connection between monocytes and macrophages. Briefly, inflammatory monocytes were recruited during infection and could differentiate into macrophages upon migration outside the vasculature (56). We measured the percentage of monocytes and macrophages in the PLF separately. Compared with the control, the proportion of monocytes in mice challenged with SEZ was increased. Furthermore, the proportion of monocytes in the GSDMD^+/+^ mice was significantly lower than the proportion in the GSDMD^-/-^ mice after SEZ infection, which suggested the existence of GSDMD-dependent pathways for inhibiting monocytes recruitment during SEZ infection (**Figure 3A**). However, compared with the control, the proportion of peritoneal macrophages was substantially elevated after SEZ infection in GSDMD^+/+^ and GSDMD^-/-^ mice (**Figure 3B**). Notwithstanding, no significant differences in the proportion of peritoneal macrophages were observed between the GSDMD^+/+^ and GSDMD^-/-^ mice infected with SEZ (**Figure 3C**). According to previous results of our laboratory (25), the number of neutrophils and lymphocytes may be increased in PLF after SEZ infection. Therefore, we speculate that GSDMD does not affect macrophage recruitment but rather macrophage function.

As previously studied, IL-18 could cooperate with IL-12 and then stimulate T helper 1 (Th1) cells-mediated immune responses against microbial infections, and subsequent studies suggested that IL-18 stimulated both innate and acquired immunity (57). IL-18 played a vital role in host defense against various infectious microorganisms because it strongly enhanced the induction of IFN-γ, nitric oxide (NO), and ROS in phagocytes (58). In addition, IL-1β release protected the host during the group A streptococcal infection (59, 60). The studies prompted us to investigate the secretion of IL-1β and IL-18 after SEZ infection. The results indicated that both IL-1β and IL-18 were released in a GSDMD-dependent manner in the PLF (**Figures 4A, B**), which suggested that intraperitoneal inoculation of SEZ induced the secretion of IL-1β and IL-18 compared with intranasal inoculation (22), playing an important role in restriction of SEZ spreading (61, 62). According to our previous results (22), SEZ infection induced lung injury, pneumonia, and more neutrophils clearing SEZ in intranasal inoculation, revealing how the host responds to pneumonia caused by SEZ. Nevertheless, more monocytes and macrophages were induced in PLF (**Figures 3A, C**) in intraperitoneal inoculation of SEZ, which may contribute to the release of IL-1β and IL-18, suggesting the host may restrict systemic infection of SEZ in the mice model of bacterial peritonitis. Combining the results of macrophages proportion (**Figure 3C**), we hypothesized that the release of IL-1β and IL-18 was mainly responsible for peritoneal macrophages. Subsequently, we found that the release of IL-1β and IL-18 in macrophage supernatants was consistent with the results of PLF (**Figure 4**). These results demonstrated that macrophages regulated the release of IL-1β and IL-18 mainly through a GSDMD-dependent manner after infection with SEZ. Previous studies have shown that GSDMD, an executor of macrophage pyroptosis, regulated the release of IL-1β and IL-18 by forming pores in the cell membrane (63, 64). We hypothesized that macrophages were induced to undergo GSDMD-mediated pyroptosis after SEZ infection. SEZ infection increased the LDH release in macrophages supernatant, but GSDMD deficiency could significantly reduce the LDH release (**Figure 5A**). The expression content of GSDMD and GSDMD-N protein in the GSDMD^+/+^ macrophages stimulated with SEZ was significantly higher than in control (**Figure 5B**), which indicated that GSDMD was cleaved to expose the GSDMD-N domain. Besides, more PI-positive cells were observed after SEZ infection, whereas the number of PI-positive cells depended on GSDMD (**Figure 6A**). Cell supernatant LDH assay, cleavage of GSDMD, and PI staining assay are usually used to detect pyroptosis, and morphology changes of pyroptotic cells can be observed by scanning electron microscopy experiments (31, 32). Ultrastructures changes of macrophages were observed after SEZ infection, including cells bubbling. However, the morphology of GSDMD^-/-^ macrophages has no significant difference between the infected and non-infected macrophages (**Figure 6B**). These results suggest the existence of GSDMD-dependent pathways for macrophages pyroptosis.

Pyroptosis has been reported to provide protection through several mechanisms. One of the most critical mechanisms was that pyroptotic cells could release IL-1β and IL-18 through the membrane pores, amplifying the inflammatory response and improving anti-infective capacity (51). We found that SEZ infection could increase IL-1β and IL-18 release *in vivo*. In contrast, GSDMD deficiency would inhibit the release of IL-1β and IL-18 after the infection (**Figure 4**). Our data suggested that macrophages may be the cellular source of the mature IL-1β and IL-18 *in vivo*. These cytokines were released in a GSDMD-dependent fashion. Thus, we speculate that the protective response absent in GSDMD^-/-^ mice (**Figure 1**) may be related to inefficient induction of IL-1β and IL-18. Furthermore, pyroptotic pores in the membrane allow various soluble antimicrobial molecules to enter the cell to kill bacteria. It is worth noting that PI-positive cells could prove pore formation, which means multiple antimicrobial molecules could kill SEZ in the pyroptotic cells. Moreover, the bubble-like protrusions could be observed in the GSDMD-dependent pathway. The small protrusion bodies formed during pyroptosis were designated as pyroptotic bodies (28), and their nature still needs further study. Taken together, our findings suggest that SEZ infection facilitated the secretion of IL-1β and IL-18 and triggered GSDMD-mediated pyroptosis in macrophages. The inflammatory response was subsequently amplified to protect against SEZ infection. These results provide essential insights into host defense mechanisms of GSDMD against SEZ, which contribute to developing therapeutics against SEZ, even Gram-positive bacterial pathogens. Whether other Gram-positive bacteria can also induce GSDMD-mediated macrophage pyroptosis needs further investigation.

## Data availability statement

The original contributions presented in the study are included in the article/Supplementary Material. Further inquiries can be directed to the corresponding author.

## Ethics statement

The animal study was reviewed and approved by the laboratory animal Monitoring Committee of Guangdong Province.

## Author contributions

GX designed and conducted the experiments, analyzed the data, and wrote the manuscript; ZG, YJL, YY, YXL, and CL were involved in experiments conducting; YH and QF funded and administrated this project and provided scientific guidance. All authors have approved the final version.

## Funding

This work was supported by (National Natural Science Foundation of China) under grant (numbers 31872443); (National Innovation and Entrepreneurship Training program of China) under grant (number 202111847019); (Guangxi Natural Science Foundation) under grant (number 2018GXNSFBA138015); (Graduate Education Innovation Plan of Guangdong Province) under grant (number 2022JGXM128); and (Academic Fund Program of Foshan University) under grant (number xsjj202209kjb06, xsjj202209kjb08, xsjj202209kjb09).

## Conflict of interest

The authors declare that the research was conducted in the absence of any commercial or financial relationships that could be construed as a potential conflict of interest.

## Publisher’s note

All claims expressed in this article are solely those of the authors and do not necessarily represent those of their affiliated organizations, or those of the publisher, the editors and the reviewers. Any product that may be evaluated in this article, or claim that may be made by its manufacturer, is not guaranteed or endorsed by the publisher.
